# Evaluating the Efficacy of Resect-and-Discard and Resect-and-Retrieve Strategies for Diminutive Colonic Polyps

**DOI:** 10.3390/life14040532

**Published:** 2024-04-21

**Authors:** Andrei Lucian Groza, Bogdan Miutescu, Cristian Tefas, Alexandru Popa, Iulia Ratiu, Roxana Sirli, Alina Popescu, Alexandru Catalin Motofelea, Marcel Tantau

**Affiliations:** 1 3rd Department of Internal Medicine, Iuliu Haţieganu University of Medicine and Pharmacy, 400012 Cluj-Napoca, Romania; groza.andrei@umfcluj.ro (A.L.G.); tefascristian@gmail.com (C.T.); matantau@gmail.com (M.T.); 2 Advanced Regional Research Center in Gastroenterology and Hepatology, Department VII: Internal Medicine II, Discipline of Gastroenterology and Hepatology, “Victor Babes” University of Medicine and Pharmacy, 300041 Timisoara, Romania; popa.alexandru@umft.ro (A.P.); ratiu.iulia@umft.ro (I.R.); sirli.roxana@umft.ro (R.S.); popescu.alina@umft.ro (A.P.); 3 Regional Institute of Gastroenterology and Hepatology “Prof. Dr. Octavian Fodor”, 400162 Cluj-Napoca, Romania; 4 Department of Internal Medicine, Faculty of Medicine, “Victor Babeș” University of Medicine and Pharmacy, 300041 Timisoara, Romania; alexandru.motofelea@umft.ro

**Keywords:** colorectal cancer, colorectal surgery, gastroenterology, colon polyps

## Abstract

Background and Objectives: Diminutive polyps present a unique challenge in colorectal cancer (CRC) prevention strategies. This study aims to assess the characteristics and variables of diminutive polyps in a Romanian cohort, intending to develop a combined resect-and-retrieve or resect-and-discard strategy that reduces the need for an optical diagnosis. Materials and Methods: A prospective cohort study was conducted at two endoscopy centers in Romania from July to December 2021. Adult patients undergoing colonoscopies where polyps were identified and resected were included. Endoscopic procedures employed advanced diagnostic features, including blue-light imaging (BLI) and narrow-band imaging (NBI). Logistic regression analysis was utilized to determine factors impacting the probability of adenomatous polyps with high-grade dysplasia (HGD). Results: A total of 427 patients were included, with a mean age of 59.42 years (±11.19), predominantly male (60.2%). The most common indication for a colonoscopy was lower gastrointestinal symptoms (42.6%), followed by screening (28.8%). Adequate bowel preparation was achieved in 87.8% of cases. The logistic regression analysis revealed significant predictors of HGD in adenomatous polyps: age (OR = 1.05, 95% CI: 1.01–1.08, *p* = 0.01) and polyp size (>5 mm vs. ≤5 mm, OR = 4.4, 95% CI: 1.94–10.06, *p* < 0.001). Polyps classified as Paris IIa, Ip, and Isp were significantly more likely to harbor HGD compared to the reference group (Is), with odds ratios of 6.05, 3.68, and 2.7, respectively. Conclusions: The study elucidates significant associations between the presence of HGD in adenomatous polyps and factors such as age, polyp size, and Paris classification. These findings support the feasibility of a tailored approach in the resect-and-discard and resect-and-retrieve strategies for diminutive polyps, potentially optimizing CRC prevention and intervention practices. Further research is warranted to validate these strategies in broader clinical settings.

## 1. Introduction

Colorectal cancer (CRC) stands as a predominant health concern worldwide, ranking as a leading cause of cancer-related mortality. Annually, estimates suggest that over 1.8 million individuals are diagnosed with CRC, resulting in approximately 880,000 deaths [1]. The prognosis of CRC significantly hinges on the stage at which it is diagnosed, emphasizing the critical role of early detection and intervention. Within this context, adenomatous polyps serve as precursors to most CRC cases, undergoing a series of molecular and histological transformations prior to their malignancy [2]. The unchecked progression from benign polyps to invasive carcinomas highlights the necessity for the prompt identification and excision of these precursor lesions to effectively intervene in the CRC development process. The main method of CRC screening is a colonoscopy, being the only one that can intervene in the progression from polyp to cancer. Polypectomy is its main advantage, but the related costs and risks sometimes outweigh the benefits. In addition to recommendations for selecting who should undergo a screening colonoscopy [3], a standardized strategy for selecting which of the resected polyps need a histopathological examination should also be developed.

Diminutive colonic polyps, defined as polyps measuring less than 5 mm in size, are often encountered during a routine screening and diagnostic colonoscopy. Their clinical significance lies in their potential contribution to the overall burden of CRC. Although each polyp has a low individual risk of malignant transformation, their prevalence in the population and cumulative risk over time make it an important focus of research and clinical management. Therefore, it is imperative to understand the nature and management of these polyps to effectively prevent or intervene in the development of CRC. The most accurate way to evaluate diminutive colonic polyps is a histopathological examination and most guidelines recommend the retrieval and histological examination of all resected polyps. However, this represents a considerable financial burden for the health systems considering the high prevalence of diminutive polyps, with debatable benefits regarding the prevention of CRC. Lately, the development of advanced endoscopic imaging technologies has shifted its focus towards the optical diagnosis of diminutive polyps, employing methods such as virtual chromoendoscopy, magnification, and artificial intelligence (AI)-enhanced devices. These technologies offer the potential for the real-time discrimination between neoplastic and non-neoplastic polyps, heralding a paradigm shift in polyp management strategies. Thus, two new strategies for dealing with diminutive polyps have emerged, called “diagnose and leave behind” for rectosigmoid diminutive hyperplastic polyps and “resect-and-discard” for the rest of the diminutive polyps. These strategies aim to streamline colonoscopies, reduce costs, and minimize the need for unnecessary histological evaluations.

The implementation of the resect-and-discard strategy, as recommended by the American Society for Gastrointestinal Endoscopy (ASGE) and the European Society for Gastrointestinal Endoscopy (ESGE), relies on precise visual assessment criteria including polyp size, shape, and surface features. The strategy is considered safe and effective when applied to appropriately selected polyps by experienced endoscopists following established guidelines. However, an optical diagnosis is not widely available due to technical considerations and a lack of training, and, in the situations where it is used, the level of confidence in the diagnosis is suboptimal among non-expert examiners. Recent studies have shown that only about 15% of European endoscopists use the resect-and-discard strategy mainly due to the fear of an incorrect optical diagnosis [4,5]. 

Considering these data and the fact that the main benefit of knowing the histology of diminutive polyps is the correct setting of the follow-up interval after the polypectomy, the aim of the present study was to assess the characteristics and variables associated with diminutive polyps in the target population, with the intent of developing a combined resect-and-retrieve or resect-and-discard strategy that obviates the need for an optical diagnosis. This endeavor seeks to furnish insights into the efficacious management of diminutive polyps, thereby contributing to the broader objective of optimizing CRC prevention and intervention practices.

## 2. Materials and Methods

### 2.1. Study Design and Selection Criteria

The design of the study and the reporting of the results was carried out according to the STROBE Statement (http://www.strobe-statement.org), and the checklist is available as Supplementary Material. 

This prospective cohort study was conducted across two endoscopy centers in Romania, Spitalul Clinic Județean de Urgență “Pius Brânzeu” in Timișoara and Centrul Medical Loyal in Zalău, over a six-month period from July to December 2021. The study aimed to include a comprehensive cohort of adult patients undergoing complete colonoscopies, where polyps were identified and resected endoscopically. 

The inclusion criteria were set to encompass all adult patients who were admitted for colonoscopy and had polyps detected and removed during the procedure. All participating endoscopists were required to have a minimum of 5 years of experience and an annual performance of at least 300 colonoscopies, ensuring a high standard of procedural expertise and diagnostic accuracy. We delineated clear exclusion criteria to maintain the study’s focus and integrity: patients subjected to emergency colonoscopies, those lacking a definitive indication for colonoscopy, individuals recommended for sigmoidoscopy instead of full colonoscopy, and patients with specific therapeutic indications that could confound the outcomes.

### 2.2. Endoscopic Equipment and Data Collection

Two advanced endoscopic systems were employed for the study: the Eluxeo 7000 system from Fujifilm, Tokyo, Japan and the Exera III 190 system from Olympus Co, Tokyo, Japan. These systems are equipped with cutting-edge optical diagnostic features, including blue-light imaging (BLI) for the Fujifilm system and narrow-band imaging (NBI) with near focus for the Olympus system, facilitating precise characterization and classification of detected polyps. Data collection spanned patient demographics, indication for colonoscopy, quality of bowel preparation, polyps topography, and characteristics, alongside results from histopathological examinations post-resection.

### 2.3. Statistical Analysis

Continuous variables that follow a normal distribution were presented as mean with standard deviation (SD), while non-Gaussian data were presented as median and inter-quartile range (IQR). The Shapiro–Wilk test was conducted to assess the sample distribution. The differences among groups for continuous data that follow a normal distribution were evaluated using Welch’s *t*-test for two groups or ANOVA for more than two groups. Post hoc analyses were conducted using the Bonferroni correction to adjust for multiple comparisons, when needed. For continuous data that do not follow a Gaussian distribution, we performed the Mann–Whitney U test for comparing two groups, and the Kruskal–Wallis test for comparing three or more groups. The χ^2^ test or Fisher’s exact test was used to evaluate differences across categorical data, particularly when expected cell counts were below five. Categorical data are presented as frequencies (n) and proportions (%). The statistical analyses were conducted using R Studio version 3.6.3, with the following packages: Tidyverse, Finalfit, MCGV, Stringdist, Janitor, and Hmisc.

## 3. Results

### 3.1. Background Characteristics

The total participant count was 427, with the mean age of the patients reported at 59.42 years, with a standard deviation of ±11.19 years. A notable gender disparity was observed, with males constituting 60.2% (257 out of 427) of the participants.

The indications for undergoing a colonoscopy varied widely among the patients. The most common reason cited was lower gastrointestinal tract symptoms, accounting for 42.6% (182 out of 427) of the cases. Screening purposes followed, with 28.8% (123 out of 427), indicating the proactive approach taken by a significant portion of the study population. Fecal immunochemical test (FIT)-positive screening was the least common indication, making up only 2.3% (10 out of 427) of the cases. Other notable indications included post-CRC surveillance at 7.3% (31 out of 427), post-polypectomy surveillance at 18.3% (78 out of 427), and surveillance for inflammatory bowel disease (IBD) at a minimal 0.7% (3 out of 427). The adequacy of bowel preparation was satisfactorily achieved in 87.8% (375 out of 427) of the cases, as presented in Table 1.

### 3.2. Lesion Characteristics

This analysis, based on a sample of 790 polyps, revealed significant findings, as detailed in Table 2. Age demonstrated a statistically significant association with polyp size, as evidenced by a *p*-value of less than 0.001. The mean age increased with the size of the polyps, from 58.6 years (SD = 10.9) for polyps measuring 0–3 mm, to 62.4 years (SD = 9.6) for polyps larger than 10 mm. 

The gender distribution, with 60.9% male and 39.1% female in the smallest polyp size group (0–3 mm), did not show a significant association with polyp size, indicated by a *p*-value of 0.7012. A histological analysis revealed a significant variance across different polyp sizes (*p* < 0.0012), particularly highlighting a decrease in hyperplastic polyps and an increase in adenomatous polyps with high-grade dysplasia (HGD) as size increased. Hyperplastic polyps were most common in the smallest size category (27.0%) and markedly decreased to 2.4% in polyps larger than 10 mm. Conversely, tubular adenomas with HGD were absent in the smallest size category but rose to 9.7% in polyps larger than 10 mm. 

The location and Paris classification also showed significant associations with polyp characteristics. Although the *p*-value for the location was 0.1152, the Paris classification demonstrated a strong correlation with polyp types (*p* < 0.0012), indicating that, as the complexity of the polyp’s morphology increases, so does the likelihood of encountering HGD. Lastly, the presence of HGD was significantly associated with polyp size, as larger polyps showed an increased presence of HGD (*p* < 0.0012). 

### 3.3. Risk Factor Analysis

The analysis, detailed in Table 3, involved various predictors including age, polyp size, number of polyps, Paris classification, and gender. The intercept of the regression model was significantly negative (−7.86) with a standard error (SE) of 1.26, indicating a low baseline probability of adenomatous polyps with HGD when other predictors are at their reference levels. This finding was statistically significant (*p* < 0.001).

Age was found to have a positive association with the likelihood of HGD in adenomatous polyps. Specifically, for each year’s increase in age, the odds of having polyps with HGD increased by 5% (odds ratio [OR] = 1.05, 95% CI: 1.01–1.08, *p* = 0.01), emphasizing the importance of age as a risk factor for HGD in polyps.

Polyp size showed a significant effect on the probability of a HGD presence. Polyps larger than 5 mm were 4.4 times more likely to have HGD compared to polyps 5 mm or smaller (OR = 4.4, 95% CI: 1.94–10.06, *p* < 0.001). The number of polyps was also a significant predictor, with each additional polyp increasing the odds of HGD by 18% (OR = 1.18, 95% CI: 1.00–1.39, *p* = 0.04).

**Figure 1:**
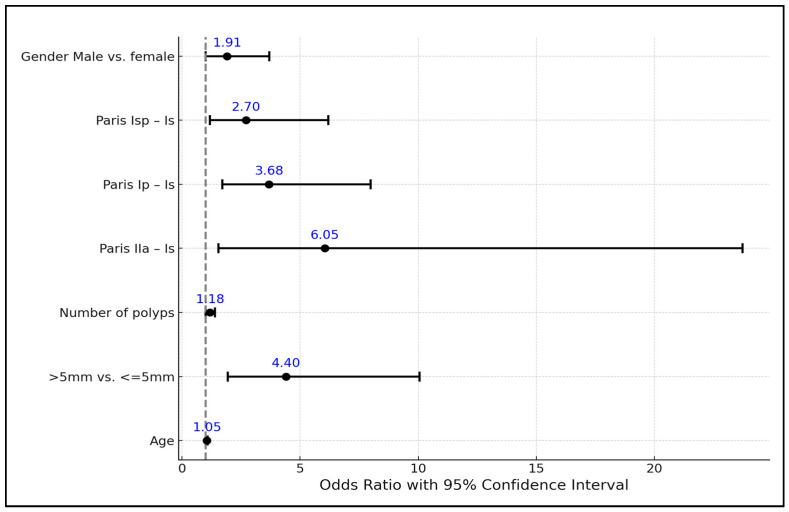
Factors impacting the probability of adenomatous polyps with high-grade dysplasia.

The Paris classification further delineated the risk, where polyps classified as IIa, Ip, and Isp were significantly more likely to harbor HGD compared to the reference group (Is). Notably, IIa polyps had the highest OR of 6.05 (95% CI: 1.54–23.73, *p* = 0.01), followed by the Ip and Isp categories, underscoring the predictive value of the polyp morphology in assessing the risk of HGD. Gender was another factor considered, with male patients having nearly twice the odds of having adenomatous polyps with HGD compared to female patients (OR = 1.91, 95% CI: 0.99–3.7, *p* = 0.05), although not statistically significant, as seen in Table 3 and Figure 1.

**Figure 2:**
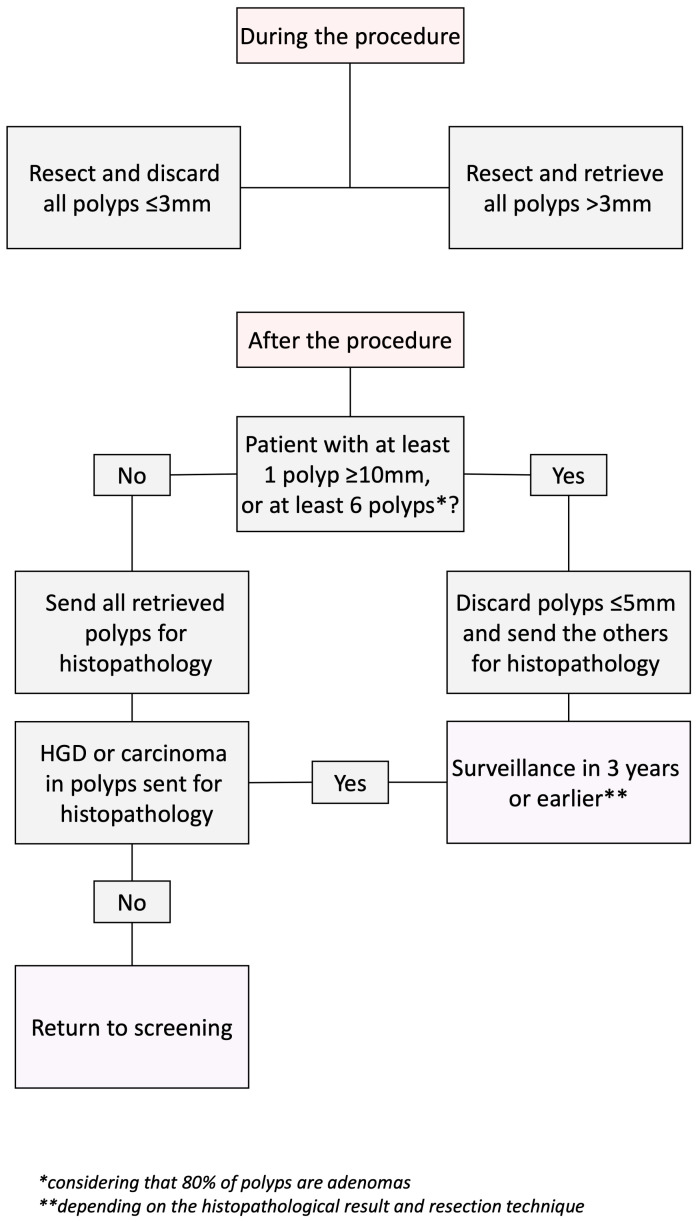
The proposed algorithm for a resect-and-discard, and a resect-and retrieve approach.

Considering all the results, we developed a post-polypectomy surveillance algorithm involving a resect-and-discard strategy without an optical diagnosis. To achieve this, we used the ESGE guideline recommendations, establishing a follow-up interval of 3 years for high-risk patients and 10 years or returning to screening for low-risk patients (Figure 2). We then compared the results obtained using this algorithm with the follow-up intervals set up after the histopathological examination. The accuracy of the strategy proposed by us to resect and retrieve or discard the diminutive polyps was 98.6% (420 of 426 patients). All six patients in whom the follow-up interval was not correctly established using the strategy proposed by us were included in the high-risk group because they had at least six resected polyps and were scheduled for follow-up at 3 years. The histopathological examination in these cases revealed less than five adenomas per patient; thus, the correct follow-up recommendation would have been returning to screening.

## 4. Discussion

Adequate bowel preparation is crucial for the correct identification of colorectal lesions during colonoscopies. The thorough cleansing of the colon ensures that the endoscopist has a clear and unobstructed view of the colonic mucosa, enabling them to detect and characterize any abnormalities, such as polyps or tumors, with greater precision. While one would believe that a better bowel preparation would yield better polyp detection, earlier studies evaluating this relationship have yielded various outcomes. Some studies found that the adenoma detection rate was not significantly different in subjects with fair bowel preparation compared to those with excellent and good bowel preparation, while others reported similar detection rates irrespective of how good or poor the preparation was [6,7,8,9]. In our study, 87.8% had an adequate bowel preparation, slightly below the ESGE recommended target of 90%, even though more than 80% of patients had a split-dosing regimen. However, as our study’s aim was to correlate optical and histopathological findings, and not quality in endoscopy, we did not consider this value to hinder our study.

Accurate polyp description using advanced endoscopic techniques is paramount in choosing the correct intervention. The most used optical classification of polyps is the NBI International Colorectal Endoscopic (NICE) Classification, based on narrow-band imaging or blue-light imaging. The classification uses vascular patterns and surface patterns to distinguish between hyperplastic and adenomatous colon polyps. NICE 1 type polyps do not require medical intervention, NICE 2 type polyps require polypectomy and monitoring, and NICE 3 type polyps require urgent treatment. In our study, only 77.3% of all polyps were correctly categorized using virtual chromoendoscopy, and this is in line with other reports in the literature [10,11]. Despite that, the diagnostic accuracy for diminutive polyps in our study was lower, at 71.6%. This could be because large, high-risk, or neoplastic lesions are more easily recognized and characterized. Regardless, this is a problem, as almost a third of the diminutive polyps in our study were incorrectly identified as hyperplastic (NICE 1), when they were adenomatous (NICE 2) in nature, and this could lead to an improper follow-up interval. 

In this respect, didactic and computer-based optical diagnosis training programs can be used to improve the accuracy of histology prediction in colorectal polyps [12]. In addition, the integration of AI in the resect-and-discard strategy for colorectal polyps is a transformative advancement in endoscopic practice. AI technologies, particularly computer-aided diagnosis systems, can analyze real-time endoscopic images and aid endoscopists, especially non-experts, in accurately differentiating between neoplastic and non-neoplastic polyps with a high sensitivity and specificity. By using machine-learning algorithms trained on extensive datasets, these systems can rapidly process and interpret visual information, providing immediate feedback during colonoscopy procedures. This not only enhances the precision of polyp characterization but also augments the overall efficiency of the resect-and-discard approach [13]. In addition, this approach could be of use where the fear of discarding polyps with advanced histology still is a significant concern. However, AI is still in its infancy in the field of endoscopy, with low adoption worldwide, mainly due to the increased implementation and maintenance costs.

A different approach could be ignoring the advanced optical diagnosis altogether and just relying on the size of the polyp. Diminutive polyps account for 75% of all polyps but rarely contain or progress to CRC [14]. The resect-and-discard strategy can be an alternative to classical histopathology for these lesions. Nonetheless, given that up to one-third of them can be optically misinterpreted, as shown above, we decided to develop a strategy that could streamline colonoscopies and polypectomies in their cases. 

The first step was to find a cut-off value for the polyp size below which all would be considered at minimal risk of malignancy. As shown in Table 2, none of the polyps measuring ≤ 3 mm harbored any HGD, while only 3.7% of those measuring 3–5 mm did. This goes to suggest that, in the case of the former, an advanced optical assessment could be considered unnecessary, as they cannot have precancerous changes. In this aspect, we suggest a laxer therapeutic approach, namely, that all these polyps could be safely resected and discarded, irrespective of number. This holds especially true in young, female patients, where we have shown that the risk of HGD is overall lower, irrespective of polyp location. Other authors have also advocated for a location-based resect-and-discard strategy, as all diminutive polyps in the rectosigmoid can be considered non-neoplastic [15]. 

Our proposed strategy should also prove to be very cost-efficient, as previously shown in other studies, as up to a third of all polyps encountered at colonoscopy would not require histological processing and interpretation [16]. In addition, these polyps can be “sized-up” quickly using a closed snare, as this generally has a diameter of 2.5 mm. Regarding the resection itself, cold snare polypectomy should be used as per the recommended guidelines, as it ensures a high rate of complete resection and low complication rates [17]. 

In the case of 3–5 mm polyps, the same plan of action could be applied in selected cases. However, as we have shown, these polyps, especially 0–Isp and 0–IIa type lesions, can harbor HGD in older, male patients. There is also a very small chance of up to 0.08% of them being cancerous, although large sample studies did not identify any cancers in diminutive as well as small polyps [18,19,20,21]. In this aspect we suggest a more prudent approach which should consider patient follow-up as well. Current guidelines recommend surveillance colonoscopy after 3 years for patients with the complete removal of at least 1 adenoma ≥10 mm or with high-grade dysplasia, or ≥5 adenomas, or any serrated polyp ≥10 mm or with dysplasia [22]. In this context, the choice to resect and retrieve or resect and discard could be based on the number and size of all lesions found during the colonoscopy. If a patient has less than five polyps and all are 3–5 mm, then these should be retrieved and the decision to either return to screening or continue surveillance should be based on the histopathological results. If, however, a patient has more than five 3–5 mm polyps or at least one ≥10 mm, all resected polyps under ≤5 mm should be discarded, as this would not change the patient’s need to undergo a surveillance colonoscopy. This should also reduce procedure-related costs. A proposed algorithm which summarizes these suggestions is found in Figure 2. Considering that the strategy proposed by us involves resecting and discarding some of the polyps, we have divided the moment when this decision is made into two stages. In the first stage, during the procedure, all polyps ≤3 mm can be resected and discarded. In this stage, polyps larger than 3 mm must be resected and retrieved. In the second, post-procedural stage, it is decided whether retrieved polyps with dimensions between 3 mm and 5 mm are sent for a histopathological examination or are discarded, depending on the other two variables: the total number of polyps and the presence of polyps larger than 1 cm. Post hoc analysis showed that the accuracy of this algorithm was 98.6%, which far exceeded the advanced optical diagnosis accuracy of 71.6% that we reported, and is even higher than the AI-assisted NBI-trained expert accuracy of 90.2% previously reported in another study as well as the proposed 90% agreement with pathology-based management according to the 2020 US Multi-Society Task Force guidelines [23,24].

The present study has some limitations. Firstly, it was a two-center experience; hence, the results need to be confirmed in further multicenter studies. Secondly, the proposed algorithm was not confirmed on other populations undergoing colonoscopies. Thirdly, the economic advantages of the resect-and-discard strategy were inferred. Even though a potential positive impact on procedure-related costs can be calculated, no precise cost analysis was performed.

## 5. Conclusions

The implementation of a size-based resect-and-retrieve or resect-and-discard strategy in our study demonstrated exceptionally high accuracy rates, surpassing those achieved through optical diagnosis and offering a significant reduction in procedure-related costs. This strategy exceeded the ≥90% benchmark agreement proposed, showcasing its potential effectiveness in clinical practice. While our findings are promising and provide a solid foundation for the efficacy of this approach, it is imperative that further studies are conducted to validate and expand upon these results. Our study underscores the potential of size-based strategies for discarding diminutive colon polyps to enhance colonoscopy efficiency and cost-effectiveness, warranting additional research to confirm these findings in broader clinical settings.

## Figures and Tables

**Table 1:** Demographic and clinical characteristics of patients.

Variables	Number of Patients (*n* = 427)
Age (years)	59.42 (±11.19)
**Gender**	
Male	257 (60.2%)
Female	170 (39.8%)
**Indication for colonoscopy**	
Lower gastrointestinal tract symptoms	182 (42.6%)
Screening	123 (28.8%)
FIT-positive screening	10 (2.3%)
Post CRC surveillance	31 (7.3%)
Post polypectomy surveillance	78 (18.3%)
IBD surveillance	3 (0.7%)
Adequate bowel preparation (%)	375 (87.8%)

FIT—Fecal Immunochemical Test; CRC—Colorectal Cancer; IBD—Inflammatory Bowel Disease.

**Table 2:** Associations between lesion characteristics and patient demographics in colorectal polyps of varied sizes.

Variables	0–3 mm (*n* = 256)	4–5 mm (*n* = 216)	6–9 mm (*n* = 153)	>10 mm (*n* = 165)	Total (*n* = 790)	*p* Value
**Age**						<0.001 ^1^
Mean (SD)	58.6 (10.9)	58.7 (10.6)	61.0 (10.5)	62.4 (9.6)	59.9 (10.6)	
Range	31.0–79.0	31.0–82.0	31.0–82.0	27.0–89.0	27.0–89.0	
**Gender**						0.701 ^2^
Female	100.0 (39.1%)	89.0 (41.2%)	54.0 (35.3%)	62.0 (37.6%)	305.0 (38.6%)	
Male	156.0 (60.9%)	127.0 (58.8%)	99.0 (64.7%)	103.0 (62.4%)	485.0 (61.4%)	
**Histology**						<0.001 ^2^
Hyperplastic	69.0 (27.0%)	20.0 (9.3%)	9.0 (5.9%)	4.0 (2.4%)	102.0 (12.9%)	
Other	1.0 (0.4%)	0.0 (0.0%)	0.0 (0.0%)	3.0 (1.8%)	4.0 (0.5%)	
SSA	14.0 (5.5%)	25.0 (11.6%)	17.0 (11.1%)	9.0 (5.5%)	65.0 (8.2%)	
TA with HGD	0.0 (0.0%)	6.0 (2.8%)	9.0 (5.9%)	16.0 (9.7%)	31.0 (3.9%)	
TA with LGD	169.0 (66.0%)	159.0 (73.6%)	106.0 (69.3%)	99.0 (60.0%)	533.0 (67.5%)	
TVA with HGD	0.0 (0.0%)	2.0 (0.9%)	3.0 (1.1%)	18.0 (10.9%)	23.0 (2.9%)	
TVA with LGD	2.0 (0.8%)	3.0 (1.4%)	8.0 (5.2%)	16.0 (9.7%)	29.0 (3.7%)	
Inflammatory	1.0 (0.4%)	1.0 (0.5%)	1.0 (0.7%)	0.0 (0.0%)	3.0 (0.4%)	
**Location**						0.115 ^2^
Right	85.0 (33.2%)	57.0 (26.4%)	48.0 (31.4%)	54.0 (32.7%)	244.0 (30.9%)	
Left	106.0 (41.4%)	102.0 (47.2%)	63.0 (41.2%)	84.0 (50.9%)	355.0 (44.9%)	
Transverse	65.0 (25.4%)	57.0 (26.4%)	42.0 (27.5%)	27.0 (16.4%)	191.0 (24.2%)	
**Paris**						<0.001 ^2^
IIa	0.0 (0.0%)	0.0 (0.0%)	1.0 (0.7%)	11.0 (6.7%)	12.0 (1.5%)	
Ip	0.0 (0.0%)	2.0 (0.9%)	14.0 (9.2%)	66.0 (40.0%)	82.0 (10.4%)	
Isp	0.0 (0.0%)	18.0 (8.3%)	38.0 (24.8%)	21.0 (12.7%)	77.0 (9.7%)	
Is	256.0 (100%)	196.0 (90.7%)	100.0 (65.3%)	67.0 (40.6%)	619.0 (78.4%)	
**HGD present**						<0.001 ^2^
No	256.0 (100%)	208.0 (96.3%)	141.0 (92.2%)	131.0 (79.4%)	736.0 (93.2%)	
Yes	0.0 (0.0%)	8.0 (3.7%)	12.0 (7.8%)	34.0 (20.6%)	54.0 (6.8%)	

^1^—Linear model Analysis of Variance (ANOVA); ^2^—Chi-square test; SD—Standard Deviation; SSA—Sessile Serrated Adenoma; TA—Tubular Adenoma; HGD—High-Grade Dysplasia; LGD—Low-Grade Dysplasia; TVA—Traditional Villous Adenoma; ANOVA—Analysis of Variance.

**Table 3:** Logistic regression analysis of factors impacting the probability of adenomatous polyps with high-grade dysplasia.

Predictor	Estimate	SE	Z	*p*-Value	Odds Ratio	95% CI—Lower	95% CI—Upper
Intercept	−7.86	1.26	−6.25	<0.001	0.001	0.001	0.004
Age	0.05	0.02	2.71	0.01	1.05	1.01	1.08
Size							
>5 mm vs. ≤5 mm	1.49	0.42	3.54	<0.001	4.4	1.94	10.06
Number of polyps	0.16	0.08	2.04	0.04	1.18	1.00	1.39
Paris:							
IIa–Is	1.8	0.7	2.58	0.01	6.05	1.54	23.73
Ip–Is	1.3	0.39	3.3	<0.001	3.68	1.7	7.98
Isp–Is	0.99	0.42	2.36	0.02	2.7	1.18	6.19
Gender							
Male vs. female	0.65	0.34	1.93	0.05	1.91	0.99	3.7

Estimates represent the log odds of “Adenoma HGD = Yes” vs. “Adenoma HGD = No”; CI—Confidence Interval; SE—Standard Error; HGD—High-Grade Dysplasia.

## Data Availability

The data are available upon request.

## Supplementary Materials

The following supporting information can be downloaded at: https://www.mdpi.com/article/10.3390/life14040532/s1. Completed STROBE checklist for cohort studies and analysis plan information.
