# Supplementary material for: Evaluating the Efficacy of Resect-and-Discard and Resect-and-Retrieve Strategies for Diminutive Colonic Polyps
Source: Life (Basel). 2024 Apr 21;14(4):532. doi: 10.3390/life14040532 (PMC11051488; doi:10.3390/life14040532)
Supplement: Supplementary file 1 [file life-14-00532-s001.zip › life-2955867-supplementary.pdf]

Completed STROBE checklist for cohort studies and analysis plan information

This checklist was elaborated using formal items recommended for cohort studies from STROBE statement (https://www.strobe-statement.org).

	Item No	Recommendation	Respected ?	Comments and quotes
**Title and abstract**	1	(*a*) Indicate the study's design with a commonly used term in the title or the abstract	Yes	Study design is indicated in the Methods/Findings section of the abstract<br>"A prospective cohort study was conducted at two endoscopy centres in Romania." Page 1 L18
		(*b*) Provide in the abstract an informative and balanced summary of what was done and what was found	Yes	These information are stated in the study abstract (study objective described, method and results described)
**Introduction**				
Background/rationale	2	Explain the scientific background and rationale for the investigation being reported	Yes	Rationale and existing literature are stated in the introduction section
Objectives	3	State specific objectives, including any prespecified hypotheses	Yes	A statement at the end of the introduction specifies the specific goals and objectives.<br>"Considering these data and the fact that the main benefit of knowing the histology of diminutive polyps is the correct setting of the follow-up interval after polypectomy, the aim of the present study was to assess the characteristics and variables associated with diminutive polyps in the target population, with the intent of developing a combined resect-and-retrieve or resect-and-discard strategy that obviates the need for op-tical diagnosis" Page 2 L85
**Methods**				
Study design	4	Present key elements of study design early in the paper	Yes	Study design is stated in the first subsection of Materials and Methods. Key elements are all described in the methods.<br>"This prospective cohort study was conducted across two endoscopy centers in Romania." Page 2 L94
Setting	5	Describe the setting, locations, and relevant dates, including periods of recruitment, exposure, follow-up, and data collection	Yes	Setting, contexts, dates of inclusion, are fully described in the Materials and Methods section under "2.1 Study Design and

				Selection Criteria" and "2.2 Endoscopic Equipment and Data Collection" headlines page 2 and 3.
Participants	6	(*a*) Give the eligibility criteria, and the sources and methods of selection of participants	Yes	Study population is described in the Materials and Methods section (2.1 Study Design and Selection Criteria) Page 3 L100
		(b) For matched studies, give matching criteria and number of exposed and unexposed	N/A	Non applicable
Variables	7	Clearly define all outcomes, exposures, predictors, potential confounders, and effect modifiers. Give diagnostic criteria, if applicable	Yes	These data can be found in Materials and Methos section (2.1 Study Design and Selection Criteria) Page 2 95
Data sources/ measurement	8*	For each variable of interest, give sources of data and details of methods of assessment (measurement). Describe comparability of assessment methods if there is more than one group	Yes	Data collection and measurement was the same for all variables, and is described in the Materials and Methods section (2.2. Endoscopic Equipment and Data Collection) Page 3 L114
Bias	9	Describe any efforts to address potential sources of bias	Yes	We notably tried to reduce bias by defining clear exclusion criteria Page 3 L104
Study size	10	Explain how the study size was arrived at	Yes	"The study aimed to include a comprehensive cohort of adult patients undergoing complete colonoscopies, where polyps were identified and resected endoscopically" Page 2 L97
Quantitative variables	11	Explain how quantitative variables were handled in the analyses. If applicable, describe which groupings were chosen and why	Yes	These are described in the Materials and Methods section under "2.3. Statistical Analysis" P3 L118
Statistical methods	12	(*a*) Describe all statistical methods, including those used to control for confounding	Yes	These are described in the Materials and Methods section under "2.3. Statistical Analysis" P3 L118
		(*b*) Describe any methods used to examine subgroups and interactions	Yes	These are described in the Materials and Methods section under "2.3. Statistical Analysis" P3 L118
		(*c*) Explain how missing data were addressed	Yes	These are described in the Materials and Methods section under "2.3. Statistical Analysis" P3 L118
		(*d*) If applicable, describe analytical methods taking account of sampling strategy	N/A	Non applicable
		(*e*) Describe any sensitivity analyses	N/A	Non applicable

**Results**				
Participants	13*	(a) Report numbers of individuals at each stage of study—eg numbers potentially eligible, examined for eligibility, confirmed eligible, included in the study, completing follow-up, and analysed	Yes	This is described at the beginning of result section "The total participant count was 427." Page 3 L132
		(b) Give reasons for non-participation at each stage	N/A	Not applicable
		(c) Consider use of a flow diagram	N/A	Use of a flow diagram was not deemed appropriate
Descriptive data	14*	(a) Give characteristics of study participants (eg demographic, clinical, social) and information on exposures and potential confounders	Yes	Table 1 describes the characteristics of the participants "Table 1. Demographic and clinical characteristics of patients." page 3 L145
		(b) Indicate number of participants with missing data for each variable of interest	N/A	Not applicable
Outcome data	15*	Report numbers of outcome events or summary measures	Yes	All numbers are reported in Tables
Main results	16	(*a*) Give unadjusted estimates and, if applicable, confounder-adjusted estimates and their precision (eg, 95% confidence interval). Make clear which confounders were adjusted for and why they were included	Yes	"Table 3. Logistic regression analysis of factors impacting the probability of adenomatous polyps with high-grade dysplasia." Page 5 L207
		(*b*) Report category boundaries when continuous variables were categorized	Yes	Category boundaries are displayed in variable headings in the tables, where applicable
		(*c*) If relevant, consider translating estimates of relative risk into absolute risk for a meaningful time period	N/A	N/A
Other analyses	17	Report other analyses done—eg analyses of subgroups and interactions, and sensitivity analyses	Yes	"Table 3. Logistic regression analysis of factors impacting the probability of adenomatous polyps with high-grade dysplasia." Page 5 L207
**Discussion**				
Key results	18	Summarise key results with reference to study objectives	Yes	Key results are described in the Discussions section, Page 8-9 L279-306, and in Conclusions.
Limitations	19	Discuss limitations of the study, taking into account sources of potential bias or imprecision. Discuss both direction and magnitude of any potential bias	Yes	Description of limitations is done under Discussions section Page 8 L308

Interpretation	20	Give a cautious overall interpretation of results considering objectives, limitations, multiplicity of analyses, results from similar studies, and other relevant evidence	Yes	In the Discussions section references were added and discussed, and, limitations were taken into account.
Generalisability	21	Discuss the generalisability (external validity) of the study results	Yes	“This strategy exceeded the ≥90% benchmark agreement proposed, showcasing its potential effectiveness in clinical practice. While our findings are promising and provide a solid foundation for the efficacy of this approach, it is imperative to conduct further studies to validate and expand upon these results.” Page 9 L321
**Other information**				
Funding	22	Give the source of funding and the role of the funders for the present study and, if applicable, for the original study on which the present article is based	Yes	Funding information are displayed at the end of the Manuscript, Page 10 L334
